# Comparative analysis of ${\dot{V}O}_{2}$ prediction equations using a novel web-based application: an illustrative example in formerly deployed military veterans

**DOI:** 10.3389/fphys.2026.1771831

**Published:** 2026-03-12

**Authors:** Thomas Alexander, Michael J. Falvo, Daniel P. Wilhite, John J. Osterholzer, Bradley W. Richmond, Steven J. Cassady, Daniel J. Schneider, Silpa Krefft, Danielle R. Glick, Anays M. Sotolongo, Stella E. Hines, Mehrdad Arjomandi

**Affiliations:** 1 Airborne Hazards and Burn Pits Center of Excellence, VA New Jersey Health Care System, East Orange, NJ, United States; 2 Research Service and Pulmonary Section Medical Service, Veterans Affairs Ann Arbor Health System, Ann Arbor, MI, United States; 3 Division of Pulmonary and Critical Care Medicine, Department of Internal Medicine, University of Michigan, Ann Arbor, MI, United States; 4 Medical Service, VA Tennessee Valley Healthcare System-Nashville, Nashville, TN, United States; 5 Department of Medicine, Division of Allergy, Pulmonary, and Critical Care Medicine, Vanderbilt University Medical Center, Nashville, TN, United States; 6 Department of Medicine, VA Maryland Healthcare System, Baltimore Veterans Affairs Medical Center, Baltimore, MD, United States; 7 National Jewish Health; Medical Service, VA Eastern Colorado Health Care System, Aurora, CO, United States; 8 Medical Service, San Francisco VA Health Care System; Department of Medicine, University of California San Francisco, San Francisco, CA, United States

**Keywords:** application (app), cardiopulmonary exercise testing, exercise capacity, prediction equations, veterans health

## Abstract

**Background:**

Cardiopulmonary exercise tests (CPET) use clinical-algorithms for interpretation by classifying exercise capacity based on a fixed threshold (e.g., oxygen consumption percent-predicted ≥80% [ $\dot{V}O_{2\text{peak}-\text{pp}}$ ]). Impact of prediction equation selection on $\dot{V}O_{2\text{peak}-\text{pp}}$ values and subsequent classifications have not been thoroughly examined in Veterans undergoing specialty evaluation for post-deployment concerns. We developed an application (https://tom26alex-cpx-comparison.share.connect.posit.cloud/) offering a direct comparison of multiple prediction equations for $\dot{V}O_{2}$ with data visualizations to better contextualize the individuals achieved ${\dot{V}O}_{2peak}$ .

**Methods:**

We retrospectively reviewed CPET records from U.S. Veterans undergoing evaluation for post-deployment concerns and calculated $\dot{V}O_{2\text{peak}-\text{pp}}$ using six separate commonly used prediction equations. Exercise capacity was classified as normal using a fixed threshold ( $\dot{V}O_{2\text{peak}-\text{pp}}$ ≥80%). Friedman’s test was employed for overall comparison of peak predicted across equations, followed by Cohen’s kappa (κ) to evaluate agreement in exercise capacity classification. The influence of demographic and anthropometric factors on inter-equation differences was examined using regression analysis.

**Results:**

Significant variability was noted in $\dot{V}O_{2\text{peak}-\text{pp}}$ between prediction equations (Friedman’s χ2 = 936.0, p < 0.01 , Kendall effect size = 0.6). In pairwise analysis, 53% of Veterans in the study were re-classified at least once resulting in significant discordance between all pairs of equations (κ = 0.24–0.78). Regression analysis identified body mass index (BMI) as the most significant contributor to differences in $\dot{V}O_{2\text{peak}-\text{pp}}$ . Given these results the app created focuses on the effects of BMI on equations by providing a visual aid to interpret the effect of BMI changes on the predicted ${\dot{V}O}_{2peak}$ .

**Interpretation:**

Classification of exercise capacity varies considerably as a function of prediction equations, and this variation appears most influenced by anthropometric factors. Clinicians should be aware of this variability and consider alternatives to relying on a single prediction equation approach, such as, utilizing the developed app to visualize and calculate a range of ${\dot{V}O}_{2peak}$ _-pp_ values derived from multiple equations.

## Introduction

An individual’s cardiorespiratory fitness, as measured by peak oxygen consumption ( ${\dot{V}O}_{2peak}$ ) during cardiopulmonary exercise testing (CPET), is recommended (American Thoracic Society/American College of Chest Physicians, 2003) to be expressed as percentage of predicted $\left(\dot{V}O_{2\text{peak}-\text{pp}}=\left[\text{measured }\dot{V}O_{2\text{peak}}/\text{predicted }\dot{V}O_{2\text{peak}}\right]*\;100\right)$ . Doing so directly influences clinical decision-making by providing a basis for understanding normal values and, should ${\dot{V}O}_{2peak}$ is reduced, serves as the starting point for interpreting diminished exercise capacity. However, many prediction equations commonly utilized were derived from relatively small (n < 100), homogeneous, predominantly male samples that were published in the 1970s. The application of these prediction equations to present populations who are generally less physically active and tested on modern CPET equipment has recently been questioned (Stickland et al., 2022). Unsurprisingly, considerable variability exists in predicted ${\dot{V}O}_{2peak}$ when using different prediction equations particularly for ethnically and racially diverse populations (Sayar et al., 2024; Shenoy et al., 2012; Braga et al., 2024), clinical samples (Brawner et al., 2018; Arena et al., 2009), and obese individuals (Busque et al., 2024).

Considerable efforts and progress have been made to improve reference standards for ${\dot{V}O}_{2peak}$ , most notably with the development of the Fitness Registry and the Importance of Exercise: A National Database (FRIEND) (Kaminsky et al., 2015) that included a large sample of men and women from geographically diverse regions across the United States using modern CPET technology. Numerous studies have compared FRIEND with other prediction equations to evaluate both diagnostic accuracy as well as prognostic importance (Braga et al., 2024; Busque et al., 2024; Chiaranda et al., 2021; Frontiers, 2024). However, these efforts in clinical populations have been largely limited to cardiovascular (e.g., heart failure, coronary artery disease) as opposed to pulmonary conditions. Given that $\dot{V}O_{2\text{peak}}$ is recommended as a primary CPET variable (Guazzi et al., 2018) for diagnostic stratification of unexplained dyspnea (c.f., Appendix 2 in Guazzi et al., 2016 (Guazzi et al., 2018)), it would appear prudent to evaluate FRIEND and other prediction equations in this context. Moreover, as obesity is a risk factor (Goh et al., 2023) for dyspnea and those with obesity are commonly evaluated for unexplained dyspnea, understanding the impact of different prediction equations on $\dot{V}O_{2\text{peak}-\text{pp}}$ is important.

To address these gaps, this study seeks to compare multiple ${\dot{V}O}_{2peak}$ prediction equations among a large sample of military veterans undergoing clinical evaluation for post-deployment health complaints, primarily unexplained dyspnea. In addition, we will determine key factors that contribute to any observed inter-equation differences. Lastly, we will describe the development and use of a custom open-source application to facilitate real-time comparison of multiple $\dot{V}O_{2\text{peak}}$ prediction equations for clinicians and researchers.

## Methods

We retrospectively reviewed clinical data from a national sample of Veterans with post-deployment health concerns who were previously deployed to the Southwest Asia Theater of Military Operations. These Veterans were evaluated at a national Department of Veterans Affairs (VA) specialty clinic (New Jersey War Related Illness and Injury Study Center) or through one of six VA’s Airborne Hazards and Burn Pits Center of Excellence’s Post-Deployment Cardiopulmonary Evaluation Network (Davis et al., 2022). Veterans included in the present study were evaluated between August 2013 to June 2024. Demographic and CPET data obtained from these assessments were included in the current study. This study was determined exempt from the Institutional Review Board (Category 4) but under the oversight of our institution’s Research & Development Committee.

### Selected peak $\dot{V}O_{2}$ prediction equations

To ensure a comprehensive comparison of predicted ${\dot{V}O}_{2peak}$ , we *a priori* selected six separate prediction equations (de Souza E Silva et al., 2018; Bruce et al., 1973; Jones et al., 1985; Hansen et al., 1984; Neder et al., 1999; Wasserman et al., 1994) based on recommendations from professional societies (American Thoracic Society/American College of Chest Physicians, 2003; Guazzi et al., 2012), as well as those that are in widespread use. Summary characteristics for each equation are described in Table 1 and additional information is available in the Online Supplement (e-Supplementary Table 1). Each patient’s measured ${\dot{V}O}_{2peak}$ was expressed relative to each of the six predicted $\dot{V}O_{2}$ values yielding a “Percent (%) predicted” value for $\dot{V}O_{2\text{peak}}\;\left(\dot{V}O_{2\text{peak}-\text{pp}}=\left[\text{Measured }\dot{V}O_{2\text{peak}}/\text{Predicted }\dot{V}O_{2\text{peak}}\right]*100\right)$ . To evaluate the impact of reference equation selection on classification of exercise capacity (i.e., normal vs. reduced), we used a conservative fixed cut-off criterion of 80% $\dot{V}O_{2\text{peak}-\text{pp}}$ .

**TABLE 1:** Characteristics of selected prediction equations complied from multiple publications.

Reference paper	Sample size (% Male)	Age (range)	BMI mean (SD)	Population used in study	Mode of testing	Protocol & methodology	Time averaging
FRIEND	10881 (68%)	20–79	26.2 (4.4)	- General Population - North American (United States) - Smoking status N/A	Bike and Treadmill	- Varied based on collection site	20–30 s
Bruce	295 (51%)	29–73	23.5 (1.4)	- General Population - North American (United States) - Included Smokers	Treadmill	- Bruce Protocol - Douglas bag	60 s
Jones	100 (50%)	15–71	23.6 (1.3)	- General Population - North American (CA) - Included Smokers	Bike	- Incremental Ramping - 16.3 W/min - Douglas bag	15 s
Wasserman	77 (100%)	34–74	29.2 (6.1)	- Shipyard workers - North American (United States) - Included Smokers	Bike	- Incremental Ramping - 30 W/min - Breath-by-breath analyzer	20 s
Hansen^a^	77 (100%)	34–74	29.2 (6.1)	- Shipyard workers - North American (United States) - Included Smokers	Bike	- Incremental Ramping - 30 W/min - Breath-by-breath analyzer	20 s
Neder	120 (50%)	20–80	26.3 (1.7)	- General Population - European (United Kingdom) - Included Smokers	Bike	- Incremental Ramping - 10–30 W/min - Breath-by-breath analyzer	15 s

^a^ The Hansen equation is a later edition of the original Wasserman equation.

BMI: body mass index, W/min: watts per minute, %: Percent, s: Seconds.

### CPET data acquisition and reduction

CPETs were performed on either a motor-driven treadmill using an incremental maximum effort protocol or cycle ergometer using a continuously increasing workload protocol. $\dot{V}O_{2}$ was measured and reported as breath-by-breath using commercially available equipment: Cosmed Quark CPET [two sites]; MGC Diagnostics Ultima PFX [three sites]; CareFusion Vyntus CPX [one site]. Testing was terminated when end-test criteria (e.g., plateau in $\dot{V}O_{2}$ with increasing workload, heart rate (HR) > 85% of age-predicted maximum, and respiratory exchange ratio >1.1) were reached as judged by the test administrator or when participants were no longer able to maintain workload despite verbal encouragement (Stickland et al., 2022). Measured ${\dot{V}O}_{2peak}$ was defined as the average $\dot{V}O_{2}$ over the last 30 s of exercise [5 sites] or the average $\dot{V}O_{2}$ over the last 8 s [1 site].

Both treadmill and cycle ergometry tests were included in the analysis, but only the Hansen and FRIEND equations had separate modality-specific equations. As treadmill protocols generally elicit higher $\dot{V}O_{2}$ values than cycle ergometry by approximately 7%–18% (Bouckaert et al., 1990), we applied a correction factor of 11% as previously implemented by Brawner and colleagues (Brawner et al., 2018). In brief, to compare cycle-to treadmill-based equations, we used a correction factor of +11%. A−11% correction factor was applied when comparing treadmill-to cycle-based equations. This correction factor was only used for equations that did not provide modality-specific equations (Supplementary e-table S1).

### Statistical analysis

For primary analysis we employed a Friedman’s test followed by an exact all-pairs comparisons with a Bonferroni adjustment to evaluate differences in $\dot{V}O_{2\text{peak}-\text{pp}}$ across equations (Pohlert, 2023). Cohen’s Kappa (κ) was used to evaluate the agreement among prediction equations for classification of exercise capacity (Gamer et al., 2019). κ reflects the frequency of ‘reclassification’ of exercise capacity–i.e., moving between classification of normal and reduced. κ values were interpreted as follows: values 0.01–0.20 as none to slight, 0.2–0.40 as fair, 0.4–0.60 as moderate, 0.61–0.80 as substantial, and 0.81–1.00 as almost perfect agreement (McHugh, 2012). Furthermore, pair-wise differences in $\dot{V}O_{2\text{peak}-\text{pp}}$ were analyzed by calculating the absolute value of the difference (AD) in $\dot{V}O_{2\text{peak}-\text{pp}}$ between each pair.

To identify key contributors to any observed differences in $\dot{V}O_{2\text{peak}-\text{pp}}$ among the equations, multivariable linear regression models were used to predict the actual difference in $\dot{V}O_{2\text{peak}-\text{pp}}$ between all 15 pairs using select variables (age, sex, weight, height, mode, and race).

Statistical significance was set at p < 0.05 and all analyses were performed using R Studio (Posit Team, 2024). The Shapiro-Wilk’s method was used to test for normality.

### R-Shiny Application

An app was built using R-Shiny (Chang et al., 2026) to assist in real-time comparison of multiple prediction equations. The app had two main goals.

1. Create an easy-to-use interface that will calculate both predicted $\dot{V}O_{2\text{peak}}$ and $\dot{V}O_{2\text{peak}-\text{pp}}$ for an individual with all six equations. The user will enter needed information to calculate the $\dot{V}O_{2\text{peak}-\text{pp}}$ (age, sex, height, weight, mode of testing, and measured $\dot{V}O_{2}$ ).
2. Plot the effects of age and weight/BMI to assist in clinical interpretations and to understand the impact of weight and age on each equation.

## Results

### Participants characteristics

Demographic and CPET data were available from 305 Veterans (Table 2). Overall, the Veterans evaluated were predominately male (89%), white (81%) and between the ages of 24–67 years of age. 60% of the CPET completed were performed on treadmill. CPET related values at peak exercise are presented in Table 3.

**TABLE 2:** Demographic information of veterans who performed a maximal effort CPET.

	N = 305
Sex	M: 89% |F: 11%
Age (years)	44 (37–51)
BMI (kg/m^2^)	31.0 (28.2–34.9)
Height (cm)	175.3 (170.2–180.3)
Weight (kg)	95.9 (85.5–110.0)
Mode of CPET	Treadmill: 60% | Bike: 40%
Endorsement of Shortness of Breath	Yes: 80%
Race	
White	81%
Black	10%
Asian	1%
Multi-Racial/Other	8%
Ethnicity	
Hispanic or Latino	10%
Non-Hispanic or Latino	82%
Unknown	8%

Median (Q1 - Q3); n (%).

CPET: cardiopulmonary exercise testing, BMI: body mass index, Q1: 25th percentile, Q3: 75th percentile.

**TABLE 3:** Peak values from maximal effort CPET.

CPET variable	N = 305
Mode of CPET	Treadmill: 60% | Bike: 40%
Absolute Peak $\dot{V}O_{2}$ (mL/min)	2,380 (1,924–2,843)
Relative Peak $\dot{V}O_{2}$ (mL/kg/min)	24.36 (19.64–29.09)
Peak VE (L/min)	85.9 (69.8–99.0)
Peak HR (bpm)	155 (138–167)
Peak RER	1.1 (1.1–1.2)
VE/VCO_2_ Nadir	24.60 (22.10–27.71)

Median (Q1 - Q3); n (%), Peak values represent the average of the last 30 s of work.

CPET: cardiopulmonary exercise testing, Q1: 25th percentile, Q3: 75th percentile, $\dot{V}O_{2}$ : oxygen consumption, V̇CO_2_: carbon dioxide consumption, VE: minute ventilation, HR: heart rate, RER: respiratory exchange ratio.

### Inter-equations comparison

**FIGURE 1:**
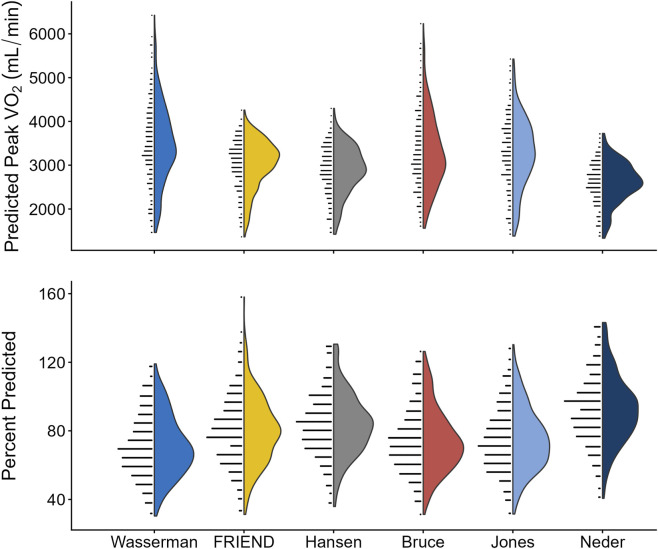
Violin plots to illustrate distribution of (top) predicted peak $\dot{V}O_{2}$ (mL/min) and (bottom) percent predicted (%) for all 6 selected reference equations. $\dot{V}O_{2}$ : Oxygen consumption.

$\dot{V}O_{2\text{peak}-\text{pp}}$ was calculated for all 305 Veterans (Table 4; Figure 1). The median measured $\dot{V}O_{2\text{peak}}$ for the sample was 2,380 mL/min (Q1-Q3: 1,924–2,843) with a range of 30%–160% predicted across all prediction equations (Figure 1). The Neder equation produced the highest mean $\dot{V}O_{2\text{peak}-\text{pp}}$ (93%) while Wasserman had the lowest (71%). Considerable variability was noted in $\dot{V}O_{2\text{peak}-\text{pp}}$ (Friedman’s χ^2^ (2) = 936.0 p < 0.01 , Kendall effect size = 0.6). Pairwise *post hoc* tests for Friedman-type showed that all pairs, except for Bruce vs. Jones ( p=0.39 ), were significantly different.

**TABLE 4:** Comparison of predicted peak $\dot{V}O_{2}$ , percent predicted, and classification of exercise capacity across prediction equations.

Reference equations	Predicted $\dot{V}O_{2}$ (mL/min)	Percent predicted (%)	Reduced exercise capacity (n (%) < 80%)
FRIEND	3,116 (2,746–3412)	80 (66, 93)	158 (52%)
Wasserman	3,439 (2,964–4,084)	70 (58, 83)	218 (71%)
Hansen	2,914 (2,551–3,318)	83 (71, 95)	128 (42%)
Bruce	3,213 (2,772–3,841)	72 (61, 85)	202 (66%)
Jones	3,277 (2,776–3,818)	73 (62, 86)	192 (63%)
Neder	2,634 (2,350–2,948)	91 (79, 103)	81 (27%)

Median (Q1 - Q3); Q1: 25th percentile, Q3: 75th percentile, $\dot{V}O_{2}$ : Oxygen Consumption.

### Classification of exercise capacity

**FIGURE 2:**
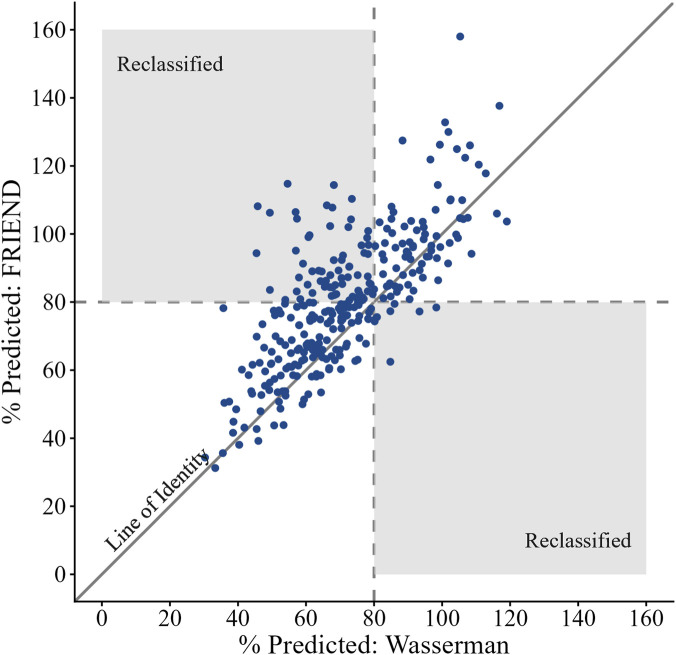
Scatterplot of the FRIEND Equation (y-axis) Percent Predicted of Peak $\dot{V}O_{2}$ Relative to Wasserman (x-axis). $\dot{V}O_{2}$ : Oxygen Consumption. Note: Dashed lines represent 80% predicted and shaded areas denote those who were re-classified into normal (upper left quadrant) or reduced (lower right quadrant) exercise capacity. The line of identity helps visualize the discrepancy between the pair of equations.

Classification of reduced exercise capacity ranged from 27% to 71% of the total cohort depending on the equation used. The Neder equation identified the fewest Veterans (n = 81, 27%) with reduced exercise capacity while the Wasserman equation classified the most (218, 71%). In this study, 53% of Veterans were reclassified at least once when comparing across all possible pairs of prediction equations. Reclassification occurred between all pairs and in both directions (Table 5). This resulted in levels of agreement among the equations from fair to substantial (κ = 0.24–0.78) with significant discordance between all pairs of equations. An inverse relationship between the median AD and κ scores (Table 5) was observed, where higher AD resulted in a higher % of Veterans being reclassified. To illustrate such a comparison, we plotted $\dot{V}O_{2\text{peak}-\text{pp}}$ calculated from the FRIEND equations against those calculated with the Wasserman equations (Figure 2). Veterans in the shaded regions on the figure denote reclassification in exercise capacity. For example, Veterans that were considered to have reduced exercise capacity per the Wasserman equation but normal exercise capacity per the FRIEND equation are seen in the top left shaded region. Of note, some Veterans, despite having higher than average AD were not reclassified. Additional comparisons between all other pairs of equations can be found in the Online Supplement (e-Supplementary Figure 1: A-N).

**TABLE 5:** Pairwise comparison of classification of exercise capacity.

From	To	Kappa	Reclassified as ‘normal’ n (%)	Reclassified as ‘reduced’ n (%)	Absolute difference (% predicted)
FRIEND	Wasserman	0.51^a^	2%	22%	8.1 (4.1–17.3)
	Hansen	0.73^a^	12%	2%	4.9 (3.1–8.6)
	Bruce	0.50^a^	5%	20%	8.9 (4.0–15.5)
	Jones	0.60^a^	5%	16%	6.9 (3.1–12.6)
	Neder	0.45^a^	27%	1%	14.6 (10.8–18.2)
Wasserman	Hansen	0.42^a^	30%	1%	11.9 (7.1–18.8)
	Bruce	0.63^a^	10%	5%	5.1 (2.7–9.5)
	Jones	0.78^a^	9%	1%	5.0 (2.4–8.0)
	Neder	0.24^a^	45%	0.3%	21.7 (16.3–27.0)
Hansen	Bruce	0.48^a^	2%	26%	9.8 (4.4–15.8)
	Jones	0.57^a^	1%	22%	8.8 (4.8–13.6)
	Neder	0.67^a^	15%	0%	8.9 (6.5–11.4)
Bruce	Jones	0.70^a^	9%	5%	4.8 (2.3–8.9)
	Neder	0.30^a^	40%	0.3%	18.3 (12.8–25.1)
Jones	Neder	0.35^a^	36%	0%	17.7 (12.7–22.9)

^a^ P < 0.001 , Median (Q1 - Q3); Q1: 25th percentile, Q3: 75th percentile.

Kappa scores presented quantifies the level of agreement in classification of exercise capacity between two pairs of equations. Percentage of Veterans reclassified and the absolute difference in percent predicted between each pair is also presented on this table.

Kappa values were interpreted as follows: values 0.01–0.20 as none to slight, 0.21–0.40 as fair, 0.41–0.60 as moderate, 0.61–0.80 as substantial, and 0.81–1.00 as almost perfect agreement.

### Factors influencing predicted $\dot{V}O_{2}$

On regression analyses, age, sex, BMI, and mode of testing were significant predictors of the actual difference of $\dot{V}O_{2\text{peak}-\text{pp}}$ in majority of the models. These select variables contributed most to the observed difference in $\dot{V}O_{2\text{peak}-\text{pp}}$ values between pairs of equations (Table 6). Notably, BMI was a statistically significant predictor for all 15 models. When comparing the FRIEND and Wasserman equations, on average, a 5-unit (1-unit changes are reported in the Online Supplement e-Supplementary Table 3) increase in BMI reflected a ∼13% increase in the difference in % predicted between FRIEND and Wasserman. Additionally, because a treadmill correction factor was applied to $\dot{V}O_{2\text{peak}-\text{pp}}$ based on mode of exercise, we conducted a sensitivity analysis by re-running all models without applying this correction. Results from the uncorrected models, included in the Online Supplement (e-Supplementary Table 2), showed similar results where BMI was the most significant predictor across all 15 models.

**TABLE 6:** Demographic and anthropometric factors’ contributions to the actual difference in percent predicted of peak $\dot{V}O_{2}$ between each pair.

From	To	Age (10 -years)	BMI (5 kg/m^2^)	Sex ref: Male	Mode ref: Bike	Race ref: White		
						Black	Asian	Other
FRIEND	Wasserman	0.94^a^	12.14^a^	−0.74	8.62^a^	−0.96	−2.55	−0.29
	Hansen	0.65^b^	4.56^a^	1.65	6.57^a^	1.03	−3.05	−1.03
	Bruce	−2.43^a^	12.94^a^	18.82^a^	8.74^a^	−1.11	−3.58	−0.59
	Jones	1.49^a^	9.28^a^	0.09	8.30^a^	−1.71	−7.16	−1.87
	Neder	1.19^a^	6.76^a^	5.63^a^	4.90^a^	1.24	−3.04	−1.70
Wasserman	Hansen	−0.30	−7.58^a^	2.39^a^	−2.05^a^	1.99^a^	−0.50	−0.74
	Bruce	−3.38^a^	0.80^a^	19.56^a^	0.12	−0.16	−1.03	−0.30
	Jones	0.54	−2.85^a^	0.82	−0.33	−0.75	−4.61	−1.58
	Neder	0.24	−5.38^a^	6.37^a^	−3.73^a^	2.20^b^	−0.49	−1.40
Hansen	Bruce	−3.08^a^	8.38^a^	17.17^a^	2.17^a^	−2.15^a^	−0.53	0.44
	Jones	0.84^b^	4.72^a^	−1.57	1.73^b^	−2.74^b^	−4.11	−0.84
	Neder	0.54^a^	2.20^a^	3.98^a^	−1.67^a^	0.21	0.01	−0.67
Bruce	Jones	3.92^a^	−3.66^a^	−18.74^a^	−0.45	−0.59	−3.58	−1.28
	Neder	3.62^a^	−6.18^a^	−13.19^a^	−3.85^a^	2.36^b^	0.54	−1.10
Jones	Neder	−0.30	−2.52^a^	5.55^a^	−3.40^a^	2.95^b^	4.12	0.17
% of Models Significant		73%	100%	67%	73%	40%	0%	0%

^a^ P < 0.001 , ^b^ P < 0.05 *,* $\dot{V}O_{2}$ : oxygen consumption, BMI: body mass index.

Each row of the table displays the parameter estimates (PE) of the demographic and anthropometric factors (i.e., groups of sex, age, BMI, and race) from regression models of the actual difference in percent predicted from one equation to another. An 11% correction factor was used for percent predicted if needed based on exercise mode.

### R-shiny for reference equation comparisons

**FIGURE 3:**
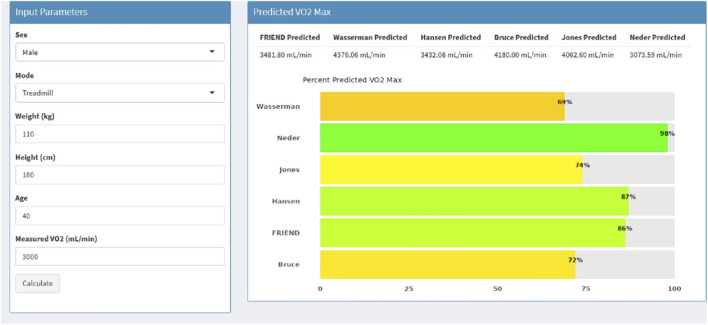
Graphical user interface of “Predicted $\dot{V}O_{2}$ Comparison” R-Shiny Application. Note: After entering input parameters, application provides predicted $\dot{V}O_{2}$ in absolute values (mL/min) and expresses measured $\dot{V}O_{2}$ as a percent predicted (dashboard).

The app named “Predicted VO2 Comparison” can be accessed here https://tom26alex-cpx-comparison.share.connect.posit.cloud/. (Additional information can be found on our GitHub repository: https://github.com/tza5051/Predicted-VO2-Comparison). Figure 3 provides an example of the dashboard created when all “input parameters” are provided.

**FIGURE 4:**
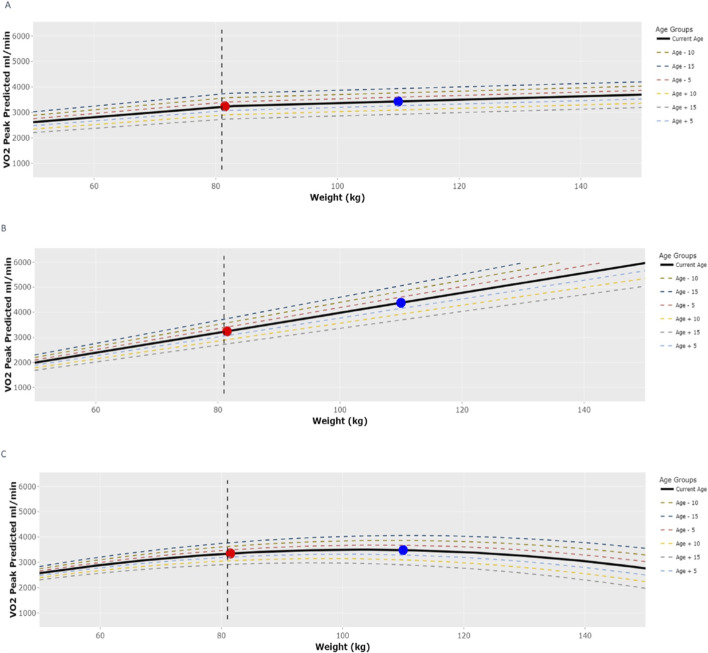
Enhanced Plotting Feature of the “Predicted $\dot{V}O_{2}$ Comparison” R-Shiny Application. **(A)** Hansen Equation, **(B)** Wasserman Equation, **(C)** FRIEND Equation. Note: Equation specific plots (**(A–C)**: Hansen, Wasserman and FRIEND) allow for visualization the influence of age and body mass on predicted $\dot{V}O_{2}$ while holding other factors (i.e., sex, height, mode, measured $\dot{V}O_{2}$ ) constant. Red and blue circles indicate ideal and actual body mass respectively.

The app provides a comprehensive assessment of $\dot{V}O_{2\text{peak}}$ and further delineates relationships between select variables (i.e., weight and age) and $\dot{V}O_{2\text{peak}-\text{pp}}$ (Figure 4). Each tab shows a graph that allows users to visualize the impact of both weight and age on the selected equations by plotting $\dot{V}O_{2\text{peak}-\text{pp}}$ at different weights (50–150 kg) and ages (±15 years) while maintaining all other variables (sex, height, mode of testing, and measured $\dot{V}O_{2}$ ) constant. Furthermore, the graph plots $\dot{V}O_{2\text{peak}-\text{pp}}$ (Figure 4) at both ideal weight (Guazzi et al., 2012) (red dot) and measured weight (blue dot). Finally, the vertical dashed line on the graph represents $\dot{V}O_{2\text{peak}-\text{pp}}$ at the BMI selected using the slider on the application.

## Discussion

This study provided a working example to illustrate the impact the selection of prediction equations has on the clinical interpretation of CPETs in a large sample of Veterans with post-deployment health complaint. In a sample of 305 formerly deployed Veterans, we found considerable discordance in exercise capacity classification between six separate prediction equations. Using a fixed threshold of 80% predicted, exercise capacity was reclassified in more than half of our sample and occurred bidirectionally (i.e., reduced to normal capacity, and normal to reduced capacity). Furthermore, the absolute magnitude of $\dot{V}O_{2\text{peak}-\text{pp}}$ between-equation differences varied by as much as 20%, suggesting reclassification was not simply due to small fluctuations of the values around the threshold of normality but a clinically meaningful difference. As noted in our analyses, patient characteristics (i.e., sex and BMI) and mode of exercise drive these differences and underscore the importance of prediction equation selection. However, any prediction equation will have its trade-offs; therefore, it may be reasonable to compare multiple equations simultaneously when evaluating an individual’s exercise capacity. To facilitate this comparison, we developed an open-source application that affords the user a direct comparison of multiple prediction equations as well as data visualization filters.

**FIGURE 5:**
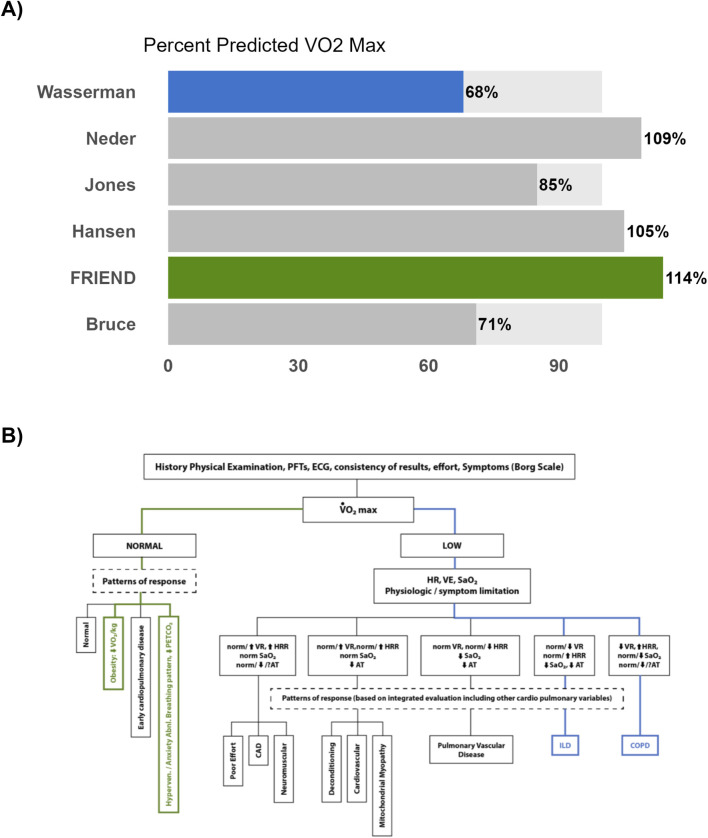
**(A)** Graphical user interface of “Predicted $\dot{V}O_{2}$ Comparison” R-Shiny Application for a 37-year-old male Veteran. **(B)** Basic strategy for the interpretation of peak CPET redrawn from ATS/ACCP (American Thoracic Society/American College of Chest Physicians, 2003) for a 37-year-old male Veteran. Note: The two potential pathways described are highlighted (Green = Pathway using FRIEND, Blue = Pathway using Wasserman). Normal $\dot{V}O_{2}$ was ≥80% predicted to stay consistent with the methods in this paper. ATS/ACCP recommends a cut-off of >84%.

Our findings may be best illustrated by a case example of a 37-year-old male Veteran (67 in, 280 lbs.) who was referred for evaluation of exertional dyspnea and other respiratory complaints believed attributable to his military environmental exposures. He underwent a maximal CPET and achieved a $\dot{V}O_{2\text{peak}}$ of 3560 mL/min (Table 7). The metabolic system software defaulted to the Wasserman predicted $\dot{V}O_{2}$ equation, yielding a $\dot{V}O_{2\text{peak}-\text{pp}}$ of 68%. According to the ATS/ACCP interpretative strategy (redrawn in Figure 5A, blue shaded line), $\dot{V}O_{2\text{peak}}$ was classified as “Low”, prompting consideration of additional CPET variables and exercise response patterns. Based on his responses, the clinician may suspect possible chronic obstructive pulmonary disease or interstitial lung disease and recommend additional testing. In contrast, if the software had applied FRIEND reference equation, the interpretation would have been quite different. Using FRIEND, his $\dot{V}O_{2\text{peak}-\text{pp}}$ was 114%, which falls within the normal range. Under this interpretation (Figure 5B, green shaded line), the schema attributes the Veteran’s exertional symptoms to factors such as obesity or anxiety. Assessment of disease severity and prognosis clearly depends on which prediction equation is presented to the clinician, and this may spell the difference between a problem that warrants in-depth investigation and one that calls for reassurance and conservative management. An alternative approach would be to consider a range of $\dot{V}O_{2\text{peak}-\text{pp}}$ values generated from multiple prediction equations. As illustrated in $\dot{V}O_{2\text{peak}-\text{pp}}$ ranges from 68% to 114% for this Veteran, with 4 out of the 6 equations classifying the Veteran with normal exercise capacity. Presenting this range could provide clinicians with greater confidence when assessing a patient’s exercise capacity and this approach has been incorporated into our application.

**TABLE 7:** Results from the CPET performed at the clinic for a 37-year-old male Veteran.

	Rest	At	VO_2_ max	Pred	VO2 max/Pred (%)
VO_2_ (mL/kg/min)	4	22.6	26.8	25.8	104
VO_2_ (L/min)	0.53	3	3.56	3.42	104
VCO_2_ (L/min)	0.42	2.94	3.61	4.14	87
Speed (MPH)		3	3		
Grade (%)		8	9		
METS	1.2	6.5	7.7	7.4	104
CARDIAC					
HR (BPM)	92	161	174	183	95
sysBP (mmHg)		184	190		
diaBP (mmHg)		67	77		
HRR (BPM)	91	22	9		
VO_2_/HR (mL/beat)	6	19	21	19	110
VENTILATION					
VE BTPS (L/min)	12.9	84.4	112.5	138	82
BR (%)	90.7	39	18.7		
Vt BTPS (L)	0.9	2.78	2.63		
RR (br/min)	14	30	43		
VE/MVV (%)	9	61	81		
GAS EXCHANGE					
PETCO2 (mmHg)	38	39	37		
PETO2 (mmHg)	98	103	108		
VE/VCO2	31	29	31	23	133
VE/VO2	24	28	32	28	112
Vd/Vt - est	0.2	0.13	0.14		
RER	0.79	0.98	1.02		
SpO_2_ (%)	97	98	99		

The Veterans is 37 years old, 280 lbs., 67 in., and performed this test on a treadmill.

AT: anerobic threshold, Pred: Predicted, %: Percent, $\dot{V}O_{2}$ : oxygen consumption, V̇CO_2_: carbon dioxide consumption, METS: metabolic equivalents, HR: heart rate, sysBP: systolic blood pressure, diaBP: diastolic blood pressure, VE: minute ventilation, BR: breathing reserve, Vt: Tidal Volume, RR: respiratory frequency, MVV: maximal ventilatory volume, PETCO_2_: Partial Pressure of End-Tidal Carbon Dioxide, PETO_2_: Partial Pressure of End-Tidal Oxygen, Vd: Dead Space, RER: respiratory exchange ratio, SpO_2_: oxygen saturation.

Quantifying the impact of $\dot{V}O_{2}$ reference equation selection has previously been considered in multiple settings (Busque et al., 2024) and among heart failure patients specifically (Brawner et al., 2018), but to our knowledge has not been specifically studied in patients undergoing evaluation for primary post-deployment concerns. Among patients with heart failure and reduced ejection fraction, Brawner et al. (2018) identified clinically meaningful differences in $\dot{V}O_{2\text{peak}-\text{pp}}$ ranging from 37% to 70% across six separate equations with an average relative $\dot{V}O_{2}$ < 16 mL∙kg^−1^∙min^−1^. Exercise capacity in our sample was much greater on average (25 mL∙kg∙min^−1^) than those with heart failure, with percent-predicted $\dot{V}O_{2}$ ranging from 70% to 90% across equations (Table 4). Still, depending on the equation, the proportion of those with reduced exercise capacity varied considerably from 27% to 71%. This discordance across equations is considerable and underscores the implications of relying on a single reference equation.

To better understand these differences, we closely examined the predictors that constitute each equation (i.e., sex, age, height, and weight) and their weighting factors. Multiple studies have emphasized the variability of $\dot{V}O_{2\text{peak}}$ predicted between different equations, particularly in ethnically and racially diverse populations (Sayar et al., 2024; Shenoy et al., 2012; Braga et al., 2024), endurance athletes (Mal et al., 2004; Jurov et al., 2023), populations with heart failure (Brawner et al., 2018; Arena et al., 2009), and populations with higher body mass index (BMI) (Busque et al., 2024). As expected, and across all equations, predicted $\dot{V}O_{2\text{peak}}$ increases with height, decreases with age, and is lower in females compared with males. Body mass, however, is treated differently across equations. Wasserman, Bruce, Jones, and Neder equations all indicate a positive linear relationship between body mass and predicted $\dot{V}O_{2\text{peak}}$ (Figure 4B), whereas FRIEND and Hansen equations are non-linear (Figures 4A,C). This non-linearity is illustrated, particularly as body mass increases, via our open-source application’s enhanced plotting feature (Figure 4). Here we utilize comparison between ideal (red dot) versus actual (blue dot) body mass for three separate equations. Through this visualization, it becomes apparent that substantial differences in predicted $\dot{V}O_{2\text{peak}}$ emerge across equations when actual body mass exceeds ideal body mass. Our analyses support these visual observations in that those equations characterized as having a non-linear relationship between $\dot{V}O_{2}$ and body mass (i.e., FRIEND and Hansen) had higher than average parameter estimates for BMI in the regression models (Table 6).

Busque et al. (2024) recently called into question the use of $\dot{V}O_{2}$ prediction equations, drawn from populations that have BMI values <30 kg/m^2^ in current clinical practice where the obesity prevalence is increasing. These authors conducted a detailed study comparing the FRIEND and Wasserman equations among patients with suspected heart failure and observed considerably reduced exercise capacity in those with obesity, and the FRIEND equation as having the greatest reduction in predicted $\dot{V}O_{2\text{peak}}$ among those who are obese. Several other groups are increasingly drawing attention to the important roles of not only body size, but body composition, to improve estimates of $\dot{V}O_{2}$ through development of new prediction equations (Santana et al., 2024). While this work is critically important to advancing the field, it may take time to fully reach clinical practice, and a one-size fits all approach to estimating $\dot{V}O_{2\text{peak}}$ may not work. Several factors influence $\dot{V}O_{2\text{peak}}$ : the influences of age, sex, body composition, mode of testing, and physical activity level on aerobic capacity is well established (Cureton et al., 1986; Toth et al., 1994).

A single equation may not be ideal since characteristics of each population are too diverse, and differences in CPET data collection hinder the possibility of pooling CPET data from different studies (Takken et al., 2019). While others advocate for development of tailored prediction equations that better reflect the specific characteristics and health conditions of the population at hand (Jeon et al., 2022), the R-Shiny: Predicted $\dot{V}O_{2}$ is a more practical and immediate solution that allows users to understand the factors that influence the equations and enable real-time comparisons for a more comprehensive analysis of $\dot{V}O_{2\text{peak}-\text{pp}}$ . We sought to develop a web-based tool for clinicians to facilitate a real-time comparison of multiple prediction equations for the purpose of enhancing their interpretations and to alleviate issues with one individual reference equation. The Supplementary section includes an example of how to use the app.

It is important to note that selections of prediction equations have implications beyond just CPET interpretations. Assessment of $\dot{V}O_{2\text{peak}}$ is not only clinically relevant but has implications for disability evaluations at the state and federal level. For example, New York state employs cutoffs based on the Hansen equation ( $\dot{V}O_{2\text{peak}-\text{pp}}$ < 85%) (Board, 2012), which, if applied to our sample, would capture 42% of Veterans from this study. Alternatively, the Department of Veterans Affairs utilizes relative $\dot{V}O_{2\text{peak}}$ (adjusted for body mass) where results <20 mL/kg/min reflects some level of disability rating (38CFR Part 4 Subpart B, 2025). Based on this cut-off, 26% (data not shown) of our Veteran sample would meet criteria for disability rating. Note that $\dot{V}O_{2\text{peak}}$ is not considered in isolation for disability evaluations, and that these examples are only intended for illustrative purposes.

### Limitations and future research

Our results should be interpreted in the contexts of our study limitations, which include primarily an analysis of cross-sectional data of a group of Veteran patients (predominantly male) undergoing specialty clinical evaluation for post-deployment health concerns. These evaluations took place in a clinical setting across multiple sites, incorporated different metabolic carts, employed different exercise test modalities (treadmill and cycle ergometer), and different post-test analysis protocol ( $\dot{V}O_{2\text{peak}}$ averaging). Despite these limitations, our primary goal was to utilize this sample as a worked example to draw attention to the clinical impact of reference equation selection. Future studies need to recognize that different prediction equations can produce substantially different $\dot{V}O_{2\text{peak}-\text{pp}}$ , and should therefore always specify the exact equation used, particularly when developing clinical interpretation algorithms in which $\dot{V}O_{2\text{peak}-\text{pp}}$ serves as the initial branching point. In addition, future work should be conducted in close collaboration with clinicians to obtain iterative feedback, improve the usability of these tools, evaluate their impact within routine clinical workflows, and determine their long-term effects on patient care.

## Interpertation

Commonly used $\dot{V}O_{2}$ prediction equations applied to our sample of symptomatic patients undergoing evaluation for post-deployment concerns yielded estimates of $\dot{V}O_{2\text{peak}-\text{pp}}$ ranging from 70% to 91%, resulting in significant discordance when classifying exercise capacity. In fact, depending on the equation, 27%–71% of our samples were classified as having reduced exercise capacity. To avoid these interpretation pitfalls and aid in clinical interpretation, we developed an open-source web-based application to facilitate real-time comparison of multiple prediction equations to better contextualize an individual’s exercise capacity.

## Data Availability

This dataset comprises clinical operational data and cannot be shared per Department of Veterans Affairs policies. There are no provisions for us to share this data publicly. Requests to access these datasets should be directed to thomas.alexander3@va.gov.
