# Supplementary material for: Comparative analysis of V˙O2 prediction equations using a novel web-based application: an illustrative example in formerly deployed military veterans
Source: Front Physiol. 2026 Mar 12;17:1771831. doi: 10.3389/fphys.2026.1771831 (PMC13017338; doi:10.3389/fphys.2026.1771831)
Supplement: Supplementary file 1 [file DataSheet1.docx]

**SUPPLEMENTAL APPENDIX**

**Comparative Analysis of V̇O_2_ Prediction Equations Using a Novel Web-Based Application: An Illustrative Example in Formerly Deployed Military Veterans.**

Table of Contents

[Supplemental Section: 3](#_Toc223430047)

[Additional Analysis and Information: 3](#_Toc223430048)

[Using the R-Shiny: Predicted V̇O2 Comparison: 3](#_Toc223430049)

[Supplemental Tables 6](#_Toc223430050)

[e-Table 1: 6](#_Toc223430051)

[e-Table 2: 7](#_Toc223430052)

[e-Table 3: 8](#_Toc223430053)

[e-Table 4: 9](#_Toc223430054)

[Figures 10](#_Toc223430055)

[e-Figure 1: 10](#_Toc223430056)

[e-Figure 2: 14](#_Toc223430057)

# **Supplemental Section:**

## **Additional Analysis and Information:**

Addition information about the Selected reference equations used to estimate V̇O_2peak_ are found on e-Table 1 including the equations used to code the R-shiny Application. Predicted values were calculated exactly as described in the original publications, with no adjustments made to match reference populations, consistent with standard practice.

Illustrative comparisons for agreement between all other pairs of equations not found in the main text can be found on e-Figure 1: A-N. All plots in this figure represent V̇O_2peak-pp_ calculated using one equation against those calculated with another to illustrate pair-wise comparison. Veterans that fall into the shaded regions (top left, bottom right) on the figure denote reclassification in exercise tolerance. Of note, not all Veterans with a high absolute difference in V̇O_2peak-pp_ were reclassified between equations. However, a change in V̇O_2peak-pp_ from 160% to 110% might have clinical implications.

Results from the sensitivity analysis where all regression models were re-run without applying the 11% correction for mode of testing are provided in e-Table 2. 1-unit changes for BMI and age are provided in e-Table 3.

## **Using the R-Shiny: Predicted V̇O2 Comparison:**

R-Shiny: Predicted V̇O_2_ Comparison allows users to not only compare the 6 equations from this study but also allows users to explore the effect of certain input variables for each equation. In this example we focus on sex by using the app to calculate V̇O_2peak-pp_ and toggling between the female and male option while keeping all other variables consent. For this example, we decided to use values based on mean values from this study (Mode = Bike, Weight = 98.17 kg, Height = 175.44 cm, Age = 44). e-Table 4 displays the different results when toggling between the two sex options. Sex had the smallest influence for the Bruce equation resulting in higher predicted values for females when compared to the other equations. This was most apparent when comparing Wasserman and Bruce. For the male Veterans in this study, the mean difference in predicted V̇O_2peak_ between the two equations was 245 mL/min (Mean V̇O_2peak_ Wasserman = 3661 mL/min, Mean V̇O_2peak_ Bruce = 3127.89 mL/min), however, the mean difference for female Veterans in our group was 455 mL/min (Mean V̇O_2peak_ Wasserman = 2071 mL/min, Mean V̇O_2peak_ Bruce = 2526 mL/min). This difference between the two equations equated to a change in V̇O_2peak_*_-_*_pp_ of about 15% for females resulting in about ~50% of females in our study to be reclassified as abnormal when utilizing Bruce instead of Wasserman.

The ‘Weight vs VO2 Graphs’ (e-Figure 2) section in the app allows users to explore the effect of BMI to the predicted V̇O_2peak_. The user can select the equation they want by selecting the different tabs above the plot. The BMI slider allows users to obtain both the predicted value and the % predicted (if a measured V̇O_2_ was provided) associated with that BMI. The slider will change the weight (vertical dashed line on plot) to achieve the selected BMI while keeping all other input parameters fixed. Furthermore, the app allows for comparison between predicted values when using ideal weight vs measured weight. By hovering the cursor over the red dot (ideal weight) and blue dot (measured weight) the user is provided with both predicted V̇O_2peak_ and V̇O_2peak_*_-_*_pp_ calculated using the selected weight.

The app also provides a peak predicted vs weight plot for each equation. This plot is further grouped based on age where the solid black line represents the age inputted by the user, while the dashed line plots predicted value based on varying ages.

# **Supplemental Tables**

## **e-Table 1:**

Selected reference equations used to estimate peak V̇O2 (mL/min).

| Reference Equations | Equations |
| --- | --- |
| Wasserman**  Hansen et al 1984: (18)  Wasserman et al. 1994: (32)   - Multiple linear regression analysis | Male:   - - weight (kg) ∗ (50.72 − (0.372 ∗ age))/1000,   Female:   - - weight (kg) + 42.8) ∗ (22.78 − (0.17 ∗ age))/1000) |
| FRIEND  Silva et al. 2018 (15)   - Multiple linear regression analysis | (45.2 – (0.35 * age) – (10.9* sex (male=1; female=2)) – (0.15 * weight (lbs)) +  (0.68 * height (in)) – (0.46 * mode (treadmill=1; bike=2)) * weight(kg) |
| Hansen  Guazzi et al. 2012 (3)   - Multiple linear regression analysis | Male:  cycle factor = 50.72 - 0.372 (age)  Ideal weight = Ideal weight (kg) = 0.79 * height (cm) - 60.7    Bike:   - - if weight < ideal: ((ideal weight + actual weight)/2) * cycle factor   - if weight = ideal: Actual Weight * Cycle Factor   - if weight > ideal: (Ideal weight * Cycle Factor) + 6 * (Actual weight - Ideal weight)   Treadmill:   - - Bike values * 1.11     Female:  cycle factor = 22.78 - 0.17 (age)  Ideal weight = Ideal weight (kg) = 0.65 * height (cm) - 42.8    Bike:   - - if weight < ideal: ((ideal weight + actual weight + 86)/2) * cycle factor   - if weight = ideal: (Actual Weight + 43) * Cycle Factor   - if weight > ideal: ((Ideal weight + 43) * Cycle Factor) + 6 * (Actual weight - Ideal Weight)     Treadmill:   - - Bike values * 1.11 |
| Bruce*  Bruce et al. 1973 (16)   - Multiple linear regression analysis | Male:   - - (60 - (0.55* age)) * weight (kg)   Female:   - - (48 - (0.37 * age) * (weight (kg)) |
| Jones**  Jones et al 1985 (17)   - Multiple linear regression analysis | Male:   - - (-3.76 + 0.034 * height (cm) + 0.022 * weight (kg) - 0.028 * age) * 1000   Female:   - - (-2.26 + 0.025 * height (cm) + 0.01 * weight (kg) - 0.018 * age) * 1000 |
| Neder**  Neder et al. 1991 (19)   - Multiple linear regression analysis | Male:   - (-24.3 * age) + (10.2 * weight (kg)) + (8.3 * height (cm)) + 1125   Female:   - (-24.3 * age) + (10.2 * weight (kg)) + (8.3 * height (cm)) + 60 |

* Developed for Treadmill testing: multiplied by 0.89 if testing was done on Bike

** Developed for Bike testing: multiplied by 1.11 if testing was done on Treadmill

## **e-Table 2:**

Demographic and Anthropometric Factors’ Contributions to the Actual Difference in Percent Predicted of Peak V̇O_2_ Between Each Pair.

| From | To | Age (10 -Years) | BMI (5 kg/m^2^) | Sex Ref: Male | Mode Ref: Bike | Race Ref: White | | |
| --- | --- | --- | --- | --- | --- | --- | --- | --- |
|  |  |  |  |  |  | **Black** | **Asian** | **Other** |
| FRIEND | Wasserman | 0.07 ^b^ | 2.53 ^a^ | -1.71 ^b^ | 0.23 | -0.79 | -2.59 | -0.76 |
|  | Hansen | 0.06 ^b^ | 0.91 ^a^ | 1.65 | 6.57 ^a^ | 1.03 | -3.05 | -1.03 |
|  | Bruce | -0.21 ^a^ | 2.53 ^a^ | 19.04 ^a^ | 1.36 ^b^ | -1.11 | -3.52 | -0.52 |
|  | Jones | 0.13 ^a^ | 1.92 ^a^ | -0.78 | -0.55 | -1.54 | -7.26 | -2.43 |
|  | Neder | 0.10 ^b^ | 1.38 ^a^ | 5.07 ^a^ | -6.01 ^a^ | 1.60 | -3.03 | -2.16 |
| Wasserman | Hansen | -0.01 | -1.62 ^a^ | 3.36 ^a^ | 6.34 ^a^ | 1.83 ^b^ | -0.46 | -0.27 |
|  | Bruce | -0.29 ^a^ | 0.01 | 20.75 ^a^ | 1.13 ^a^ | -0.32 | -0.93 | 0.24 |
|  | Jones | 0.06 | -0.61 ^a^ | 0.92 | -0.78 | -0.75 | -4.67 | -1.68 |
|  | Neder | 0.03 | -1.15 ^a^ | 6.78 ^a^ | -6.24 ^a^ | 2.39 ^b^ | -0.44 | -1.41 |
| Hansen | Bruce | -0.28 ^a^ | 1.62 ^a^ | 17.39 ^a^ | -5.21 ^a^ | -2.14 ^b^ | -0.47 | 0.51 |
|  | Jones | 0.07 | 1.01 ^a^ | -2.44 ^b^ | -7.12 ^a^ | -2.58 ^b^ | -4.21 | -1.40 |
|  | Neder | 0.03 | 0.47 ^a^ | 3.41 ^a^ | -12.58 ^a^ | 0.56 | 0.02 | -1.13 |
| Bruce | Jones | 0.35 ^a^ | -0.61 ^a^ | -19.83 ^a^ | -1.90 ^a^ | -0.43 | -3.74 | -1.92 |
|  | Neder | 0.31 ^a^ | -1.15 ^a^ | -13.97 ^a^ | -7.37 ^a^ | 2.71 ^b^ | 0.49 | -1.65 |
| Jones | Neder | -0.03 | -0.54 ^a^ | 5.85 ^a^ | -5.46 ^a^ | 3.14 | 4.23 | 0.27 |
| % of Models Significant |  | 66% | 93% | 80% | 80% | 40% | 0% | 0% |

^a^ *P* < 0.001, ^b^ *P* < 0.05*,* V̇O_2_: Oxygen Consumption, BMI: Body Mass Index Note: Each row of the table displays the parameter estimates (PE) of the demographic and anthropometric factors (i.e., groups of sex, age, BMI, and race) from regression models of the actual difference in percent predicted from one equation to another. No corrections in percent predicted were made for modality.

## **e-Table 3:**

Factors’ Contributions to the Actual Difference in Percent Predicted of Peak V̇O_2_ Between Each Pair for 1-Unit Changes to BMI and Age For Both Corrected and Uncorrected Models.

| **From** | **To** | **Age (1 -Years)** | **BMI (1 kg/m^2^)** | **Age (1 -Years)** | **BMI (1 kg/m^2^)** |
| --- | --- | --- | --- | --- | --- |
|  |  | **Uncorrected** | | **Corrected** | |
| **FRIEND** | Wasserman | 0.01 ^b^ | 0.51 ^a^ | 0.09 ^a^ | 2.43 ^a^ |
|  | Hansen | 0.01 ^b^ | 0.18^a^ | 0.07 ^b^ | 0.91 ^a^ |
|  | Bruce | -0.02 ^b^ | 0.51 ^a^ | -0.24 ^a^ | 2.59 ^a^ |
|  | Jones | 0.01 ^a^ | 0.38 ^a^ | 0.15 ^a^ | 1.86^a^ |
|  | Neder | 0.01 ^b^ | 0.28 ^a^ | 0.19 ^a^ | 1.35 ^a^ |
| **Wasserman** | Hansen | 0 | -0.32 ^a^ | -0.03 | -1.51 ^a^ |
|  | Bruce | -0.02 ^a^ | 0 | -3.38 ^a^ | 0.16 ^a^ |
|  | Jones | 0.01 | -0.12 ^a^ | 0.05 ^a^ | -0.57 ^a^ |
|  | Neder | 0 | -0.23 ^a^ | 0.02 ^a^ | -1.08 ^a^ |
| **Hansen** | Bruce | -0.03 ^a^ | 0.324 ^a^ | -0.31 ^a^ | 1.68 ^a^ |
|  | Jones | 0.01 | 0.202 ^a^ | 0.08 ^a^ | 0.94 ^a^ |
|  | Neder | 0 | 0.094 ^a^ | 0.05 ^a^ | 0.44 ^a^ |
| **Bruce** | Jones | 0.04 ^a^ | -0.122 ^a^ | 0.39 ^a^ | -0.73 ^a^ |
|  | Neder | 0.03 ^a^ | -0.23 ^a^ | 0.36 ^a^ | -1.24 ^a^ |
| **Jones** | Neder | 0 | -0.108 ^a^ | -0.03 | -0.50 ^a^ |

^a^ *P* < 0.001, ^b^ *P* < 0.05*,* V̇O_2_: Oxygen Consumption, BMI: Body Mass Index Note: Each row of the table displays the parameter estimates (PE) of the demographic and anthropometric factors from regression models of the actual difference in percent predicted from one equation to another.

## **e-Table 4:**

Effect of Sex on Predicated V̇O_2_ Peak (mL/min) collected using the R-Shiny: Predicted V̇O2 Comparison.

| Option Selected | FRIEND | Wasserman | Hansen | Bruce | Jones | Neder |
| --- | --- | --- | --- | --- | --- | --- |
| Male: | 3188.96 | 3372.34 | 2797.57 | 3127.89 | 3132.70 | 2513.29 |
| Female: | 2118.91 | 2156.84 | 1909.41 | 2771.42 | 2315.70 | 1914.69 |
| Difference | **1070.05** | **1215.5** | **888.16** | **356.47** | **817.00** | **598.60** |

Note: The application was used to calculate predicted peak V̇O_2_ at both the male and female option while keeping all other input variables the same. (Mode = Bike, Weight = 98.17, Height = 175.44, Age = 44)

# **Figures**

## **e-Figure 1:**

Scatterplots Comparing the Percent Predicted of Peak V̇O_2_ Between Two Selected Equations.

A) FRIEND vs Hansen, B) FRIEND vs Bruce, C) FRIEND vs Jones, D) FRIEND vs Neder, E) Wasserman vs Hansen, F) Wasserman vs Bruce, G) Wasserman vs Jones, H) Wasserman vs Neder, I) Hansen vs Bruce, J) Hansen vs Jones, K) Hansen vs Neder, L) Bruce vs Jones, M) Bruce vs Neder, N) Jones vs Neder

Note: Dashed lines represent 80% predicted and shaded areas denote those who were re-classified into normal (upper left quadrant) or reduced (lower right quadrant) exercise tolerance. The line of identity helps visualize the disagreement between each pair of equations.


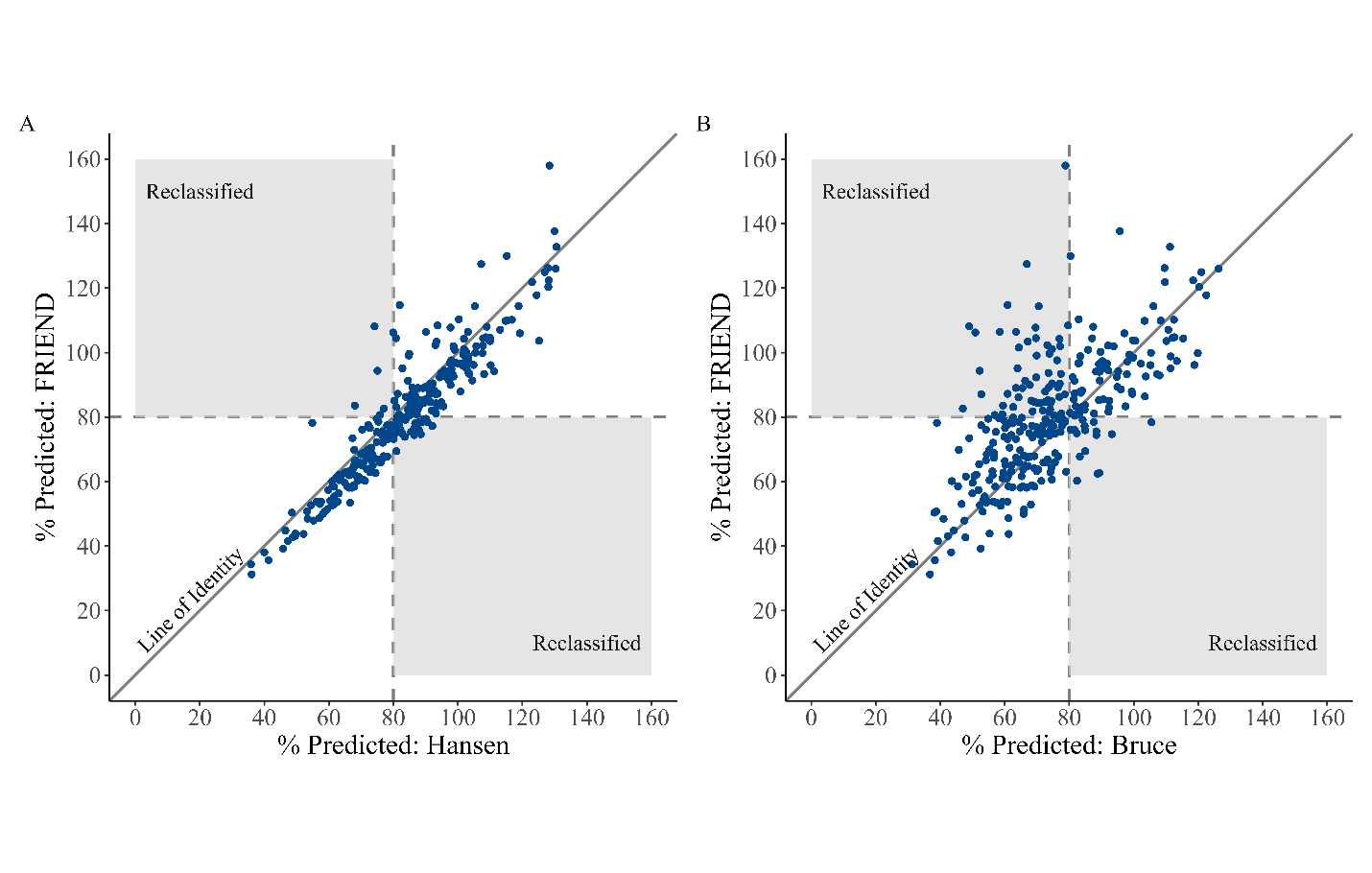


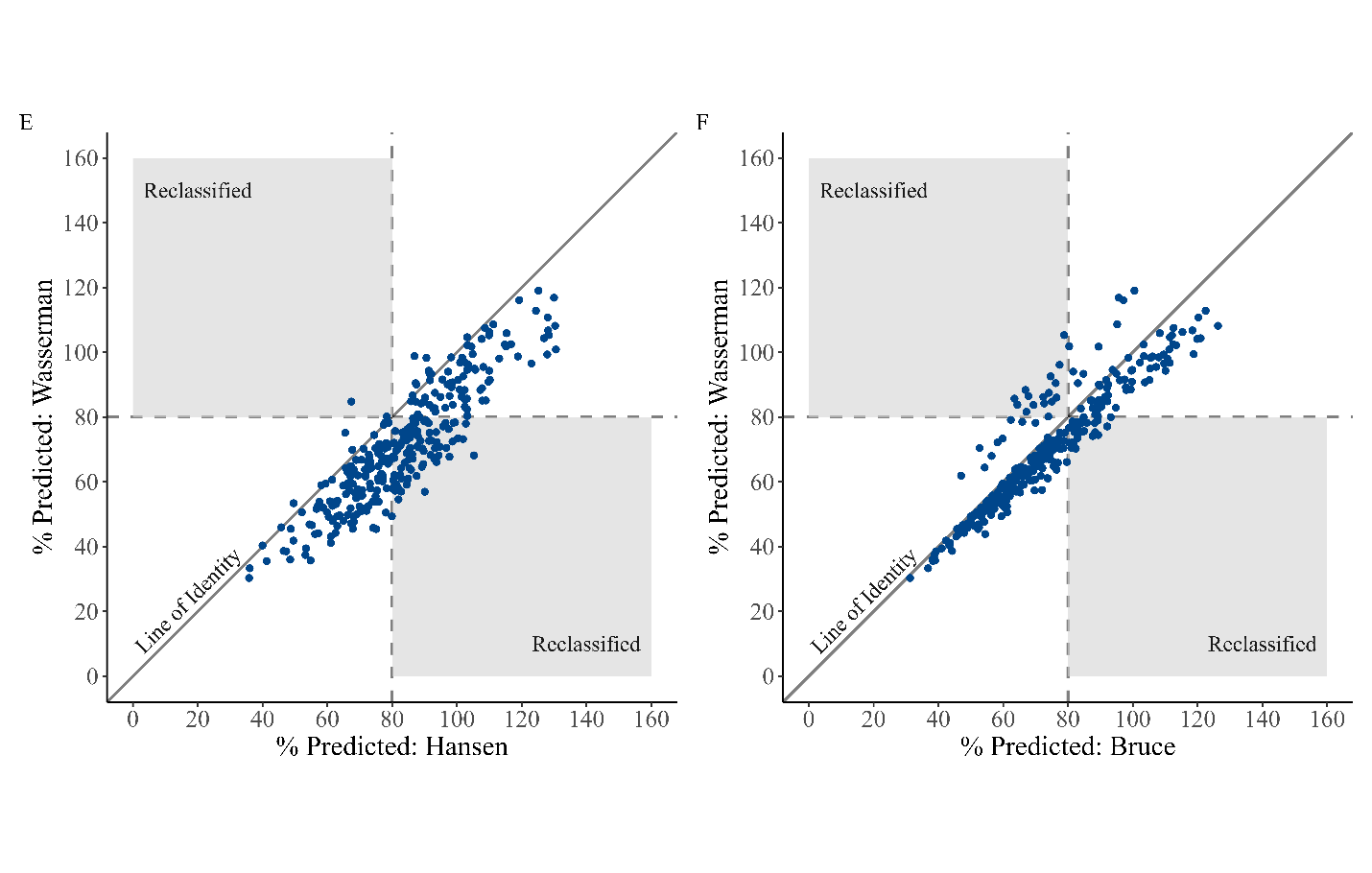

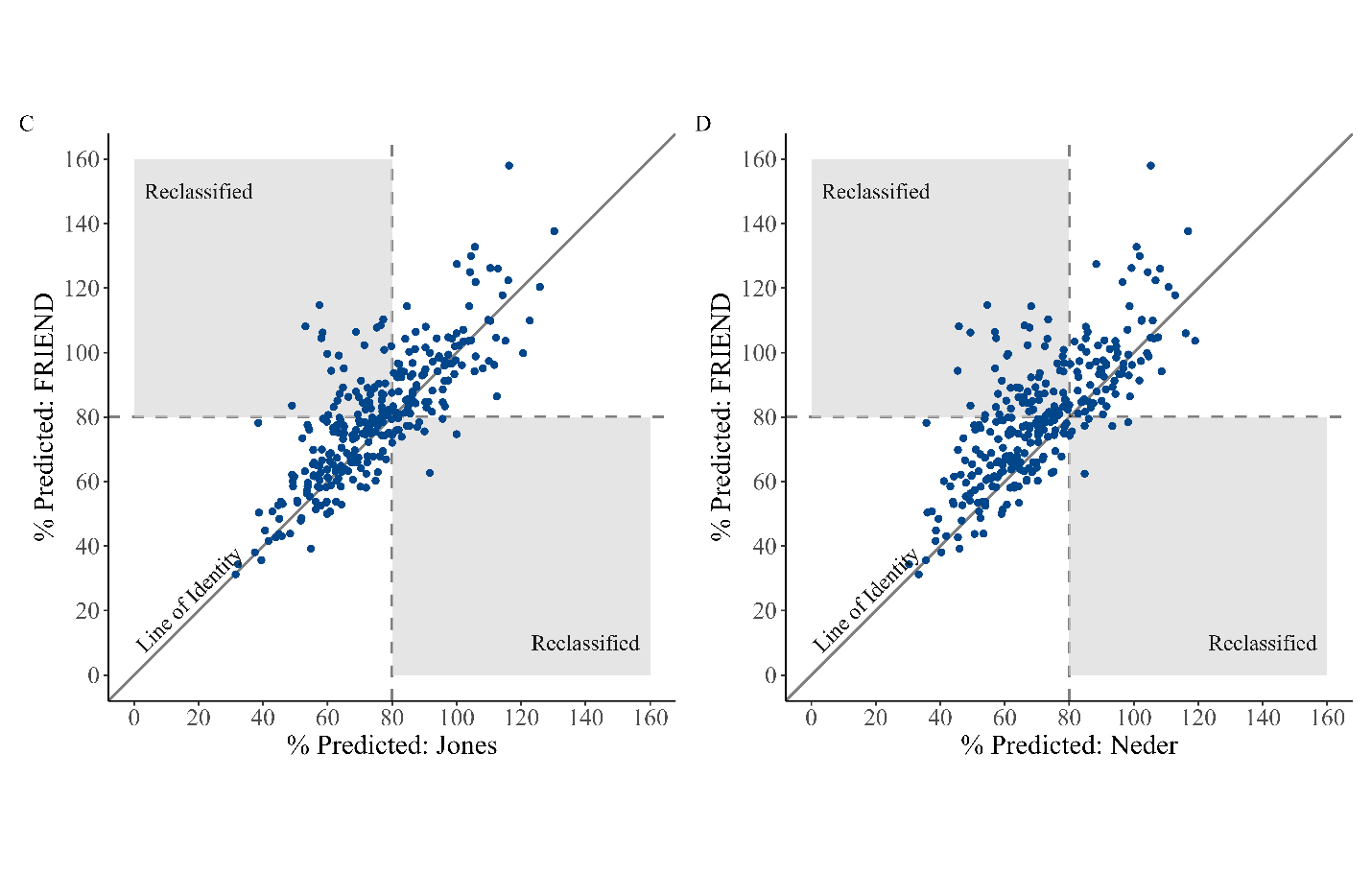


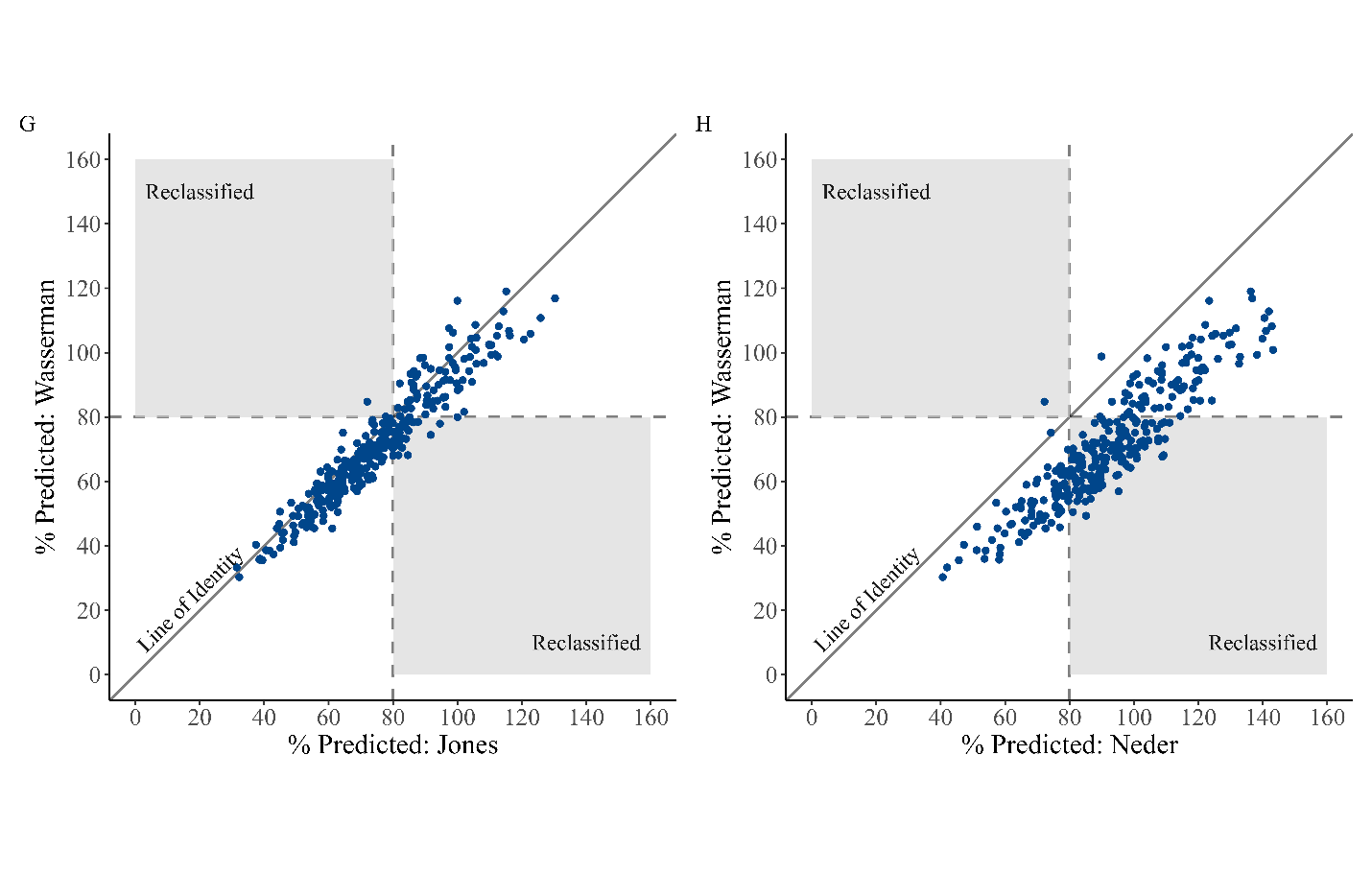


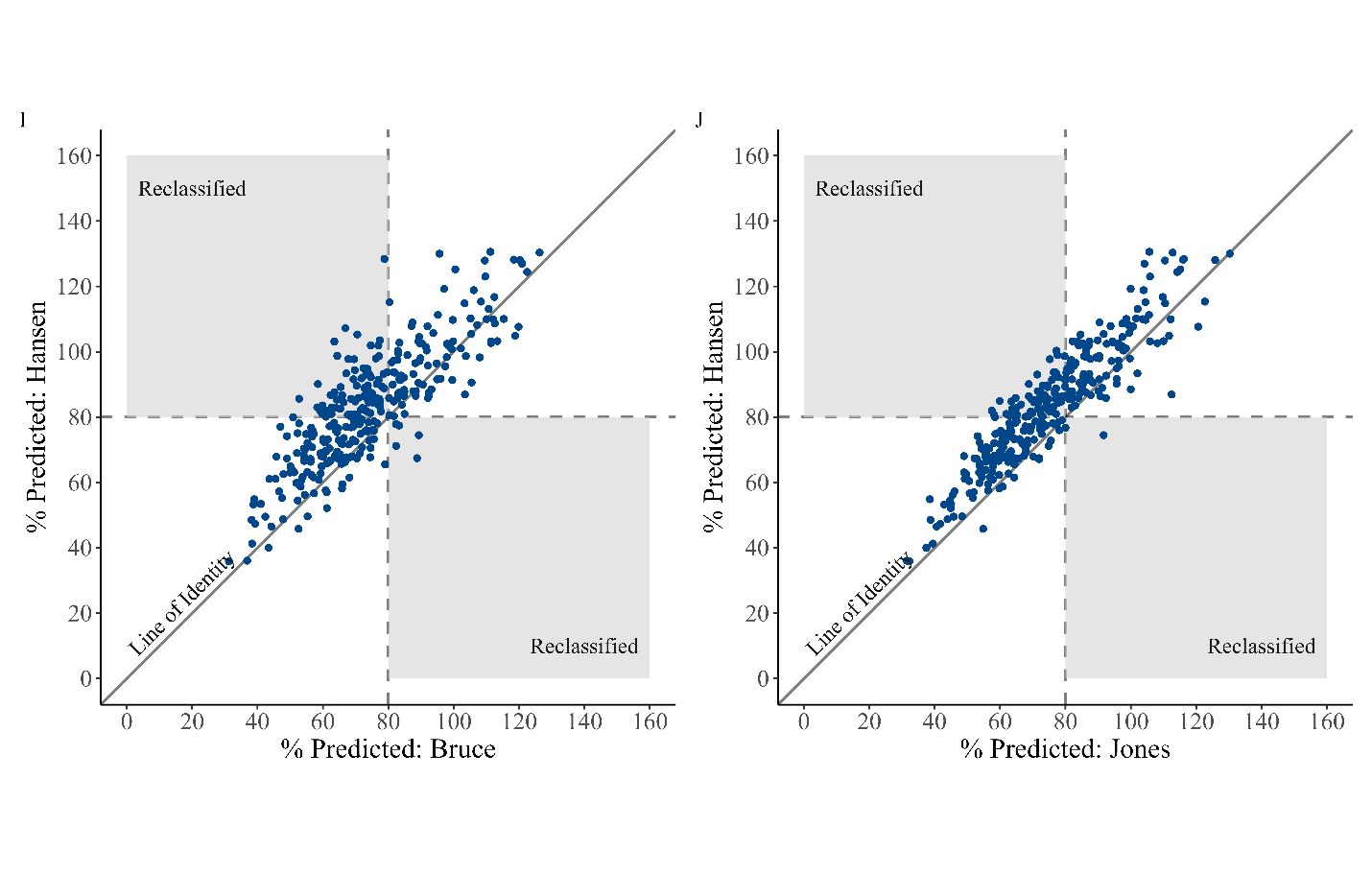


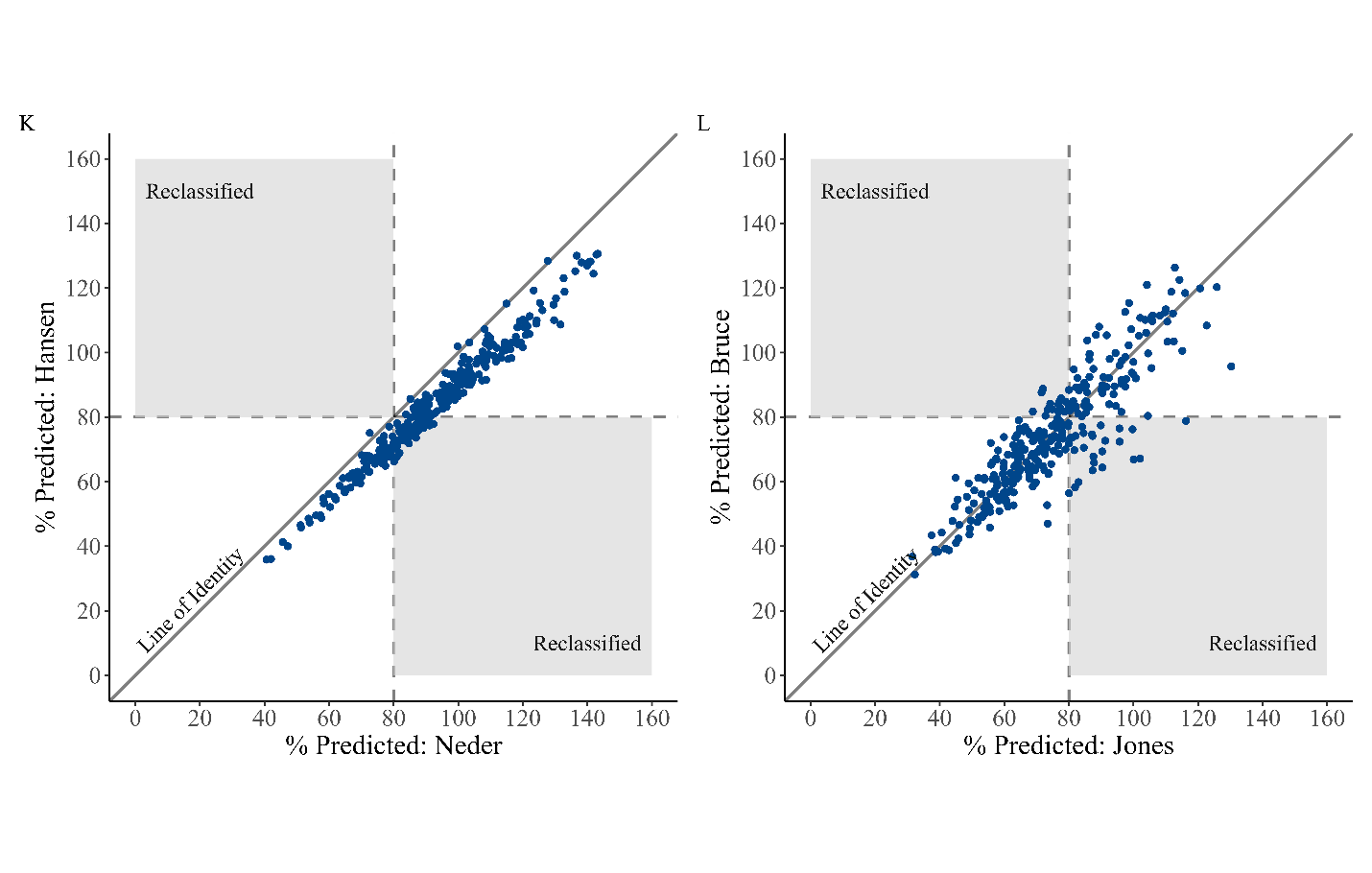


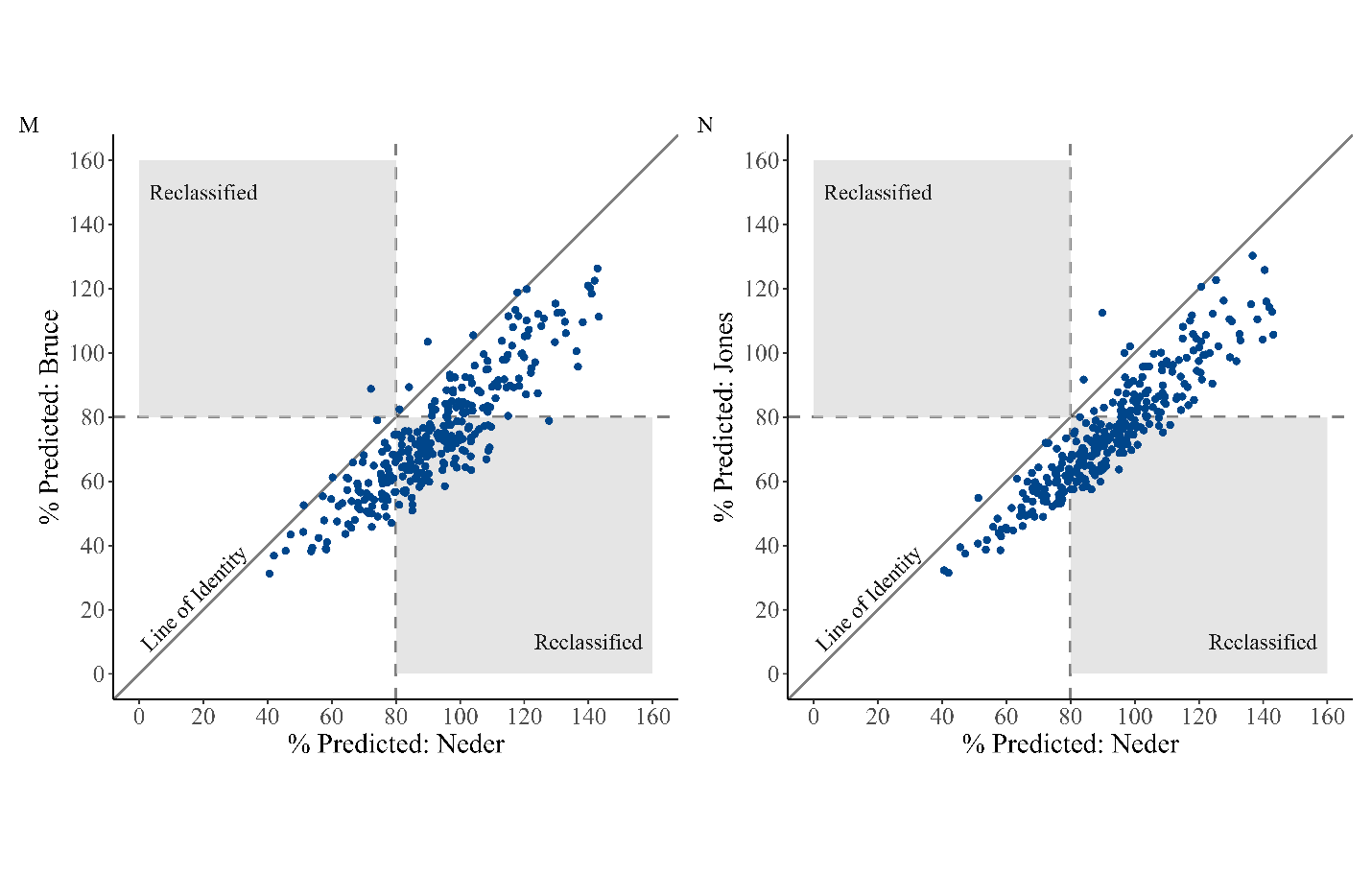


## **e-Figure 2:**

**
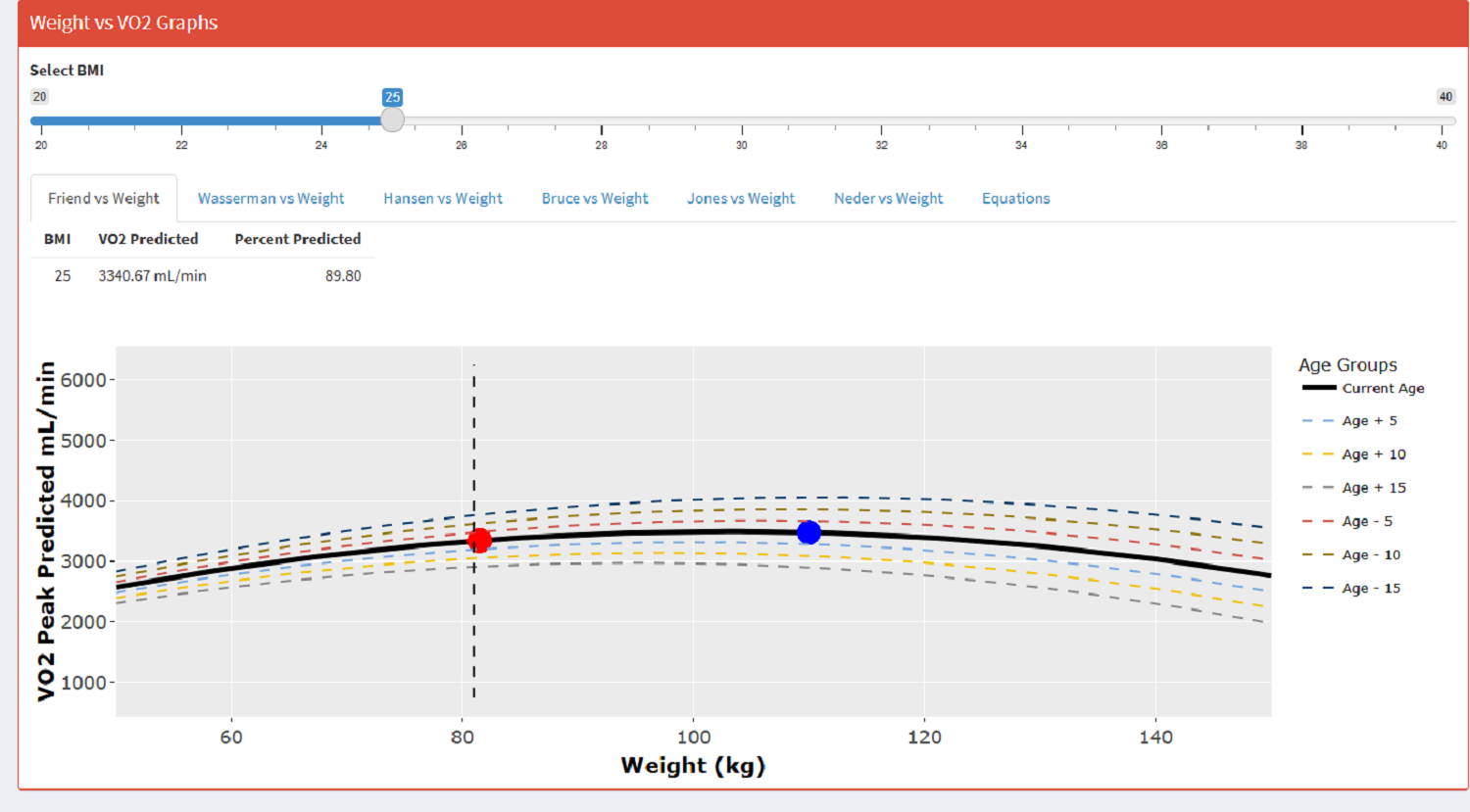
**Enhanced Plotting Feature of the ‘Predicted V̇O2 Comparison’ R-Shiny Application.
